# Sensor-Based Automated Detection of Electrosurgical Cautery States

**DOI:** 10.3390/s22155808

**Published:** 2022-08-03

**Authors:** Josh Ehrlich, Amoon Jamzad, Mark Asselin, Jessica Robin Rodgers, Martin Kaufmann, Tamas Haidegger, John Rudan, Parvin Mousavi, Gabor Fichtinger, Tamas Ungi

**Affiliations:** 1 School of Computing, Queen’s University, Kingston, ON K7L 3N6, Canada; josh.ehrlich@queensu.ca (J.E.); a.jamzad@queensu.ca (A.J.); mark.asselin@queensu.ca (M.A.); jessica.rodgers@queensu.ca (J.R.R.); mousavi@queensu.ca (P.M.); fichting@queensu.ca (G.F.); 2 Department of Surgery, Kingston Health Sciences Centre, Kingston, ON K7L 2V7, Canada; martin.kaufmann@queensu.ca (M.K.); john.rudan@kingstonhsc.ca (J.R.); 3 University Research and Innovation Center (EKIK), Óbuda University, 1034 Budapest, Hungary

**Keywords:** automated electrosurgical cautery, sensor-based parameter setting, computer-assisted surgery, robotic surgery

## Abstract

In computer-assisted surgery, it is typically required to detect when the tool comes into contact with the patient. In activated electrosurgery, this is known as the *energy event*. By continuously tracking the electrosurgical tools’ location using a navigation system, energy events can help determine locations of sensor-classified tissues. Our objective was to detect the energy event and determine the settings of electrosurgical cautery—robustly and automatically based on sensor data. This study aims to demonstrate the feasibility of using the cautery state to detect surgical incisions, without disrupting the surgical workflow. We detected current changes in the wires of the cautery device and grounding pad using non-invasive current sensors and an oscilloscope. An open-source software was implemented to apply machine learning on sensor data to detect energy events and cautery settings. Our methods classified each cautery state at an average accuracy of 95.56% across different tissue types and energy level parameters altered by surgeons during an operation. Our results demonstrate the feasibility of automatically identifying energy events during surgical incisions, which could be an important safety feature in robotic and computer-integrated surgery. This study provides a key step towards locating tissue classifications during breast cancer operations and reducing the rate of positive margins.

## 1. Introduction

Offering benefits to both patients and clinicians, robotic and computer-assisted surgeries (CAS) are becoming increasingly prevalent in the surgical suite [1,2,3,4]. One of the key technical features of CAS systems is the level of autonomy they provide [5], and another recent opportunity is to reduce the physical contact between the patient and the clinical staff [6]. The subject procedures of CAS often employ electrosurgical and power tools, which are sometimes tracked in real-time to assist robotic or navigated targeting. The data gathered shall guide the surgeon to the pre-operatively defined target anatomical locations. In CAS, it may be necessary to detect when an activated electrosurgical tool (applied part of a surgical device from the standards point of view [7]) comes into contact with the patient; this is known as the energy event [8]. For electrosurgical cautery, placing an accurate timestamp on the energy event is necessary when locating tissue classifications from an intra-operative sensor that characterizes tissue properties. By tracking the electrosurgical tools and detecting the energy events in time, a surgical navigation system can determine locations of sensor-classified tissues of interest [9]. One such sensor-based technology is the in vivo spectrometry, for which experimental devices already exist, such as the Rapid Evaporative Ionization Mass Spectrometry (REIMS), which can be physically attached to a surgeon’s electrosurgical cautery. REIMS shows a promise for improved patient outcome because it has high sensitivity and specificity scores in metabolomic tissue identification [9,10,11,12,13]. This means that the REIMS is able to distinguish cancerous from healthy tissue.

In CAS procedures, cautery tools are powered by an electrosurgical generator unit (ESU), which outputs a high-frequency alternating current transferred into thermal energy upon tissue contact. The electric current flows from the ESU to the active electrode (the cautery tip), then to the target tissue of the patient, and finally to a neutral electrode, known as the grounding pad [14,15]. During a procedure, an ESU has both “cut” and “coagulate” modes, where the former seals vessels and tissue with gradual heating, and the latter creates incisions through rapid heating.

NaviKnife is a CAS system showing promise for use in breast-conserving surgery (BCS) among other surgical domains, as it can differentiate tumors from healthy tissue and is easily incorporated into the surgical workflow. The NaviKnife system features a REIMS device attached to the surgeon’s spatially-tracked (navigated) cautery tool. REIMS generates continuous mass spectrum data from the vapor generated while the surgeon cauterizes tissue. Mass spectrum data is used to automatically detect what tissue was being cauterized within a few seconds [9,10,11,13,16,17]. Nevertheless, synchronized position tracking is required to map these tissue types on a surgical navigation display. To match position information with tissue classification, we need to know exactly the timestamp of the energy event [18].

Beyond cautery tools, it may be possible and necessary to detect energy events in other devices, such as those used in radiofrequency (RF) ablation, robotic surgery, and telesurgery. In RF ablation, tissue surrounding the ablator may become dry or charred, causing a loss of contact with the tool [19]. By tracking the ablator’s energy level throughout the procedure, this loss of contact may be detected rapidly. In robotic surgery and telesurgery, surgeons typically do not receive tactile feedback from their tools, as they interact solely with the robot’s controller. By detecting energy events, surgeons could be immediately notified when their power tools are activated and touching a patient. Robots that detect energy events may also be able to limit rapid and accidental surgical movements by performing motion scaling, motion compensation, and tremor compensation, as typical surgical sub-tasks subject to automation [20]. A prime example for such systems is the multi-degree of freedom electrosurgical tool for the smart tissue autonomous robot (STAR), developed at Johns Hopkins University (and before that at University of Maryland) [21].

Nevertheless, there are certain requirements which shall be met in order to robustly identify the energy event of surgical power tools:

1. Surgeons must activate the power tool before touching a patient, requiring the collection of both activation and contact information;
2. Any solution must be easily incorporated into the surgical workflow and cannot interfere with clinically approved devices;
3. Many surgical power tools cannot connect to a computer and display the mode or activation status. Therefore, they must be modified before they can be connected to a computer.

In previous works by Asselin et al. and Carter et al., the feasibility of using relative voltage signals to detect the energy event of a surgical cautery was demonstrated [8,22]. To achieve this, relative voltage signals were collected, and the data was clustered based on the cautery mode [8,22]. However, to implement this system in the operating room, the energy event must be detected automatically. It is also essential to identify those of the cautery’s settings, which may cause interference, such as energy level and mode. The energy level may interfere with pacemakers and other robotic devices involved in the procedure, whereas the mode may cause slight changes in the mass spectra, interfering with the classification model used by the REIMS component of NaviKnife. Further, if the CAS procedure involved intra-operative Magnetic Resonance Imaging, synchronizing the image acquisition is absolutely necessary [23].

Our objective was to detect the energy event and settings of the cautery robustly and automatically, with a sensor-based technique that does not disrupt the surgical workflow. Sufficient detection accuracy is defined as greater than 90% accuracy in detecting the energy event and settings. Key parameters expected to be determined form the sensory information:

- identification of the start and end of surgical incisions;
- cautery’s mode (cut vs. coagulate);
- cautery’s energy level.

Doing so will demonstrate the feasibility of using non-invasive current sensors to detect the cautery state to identify the exact time of surgical incisions.

## 2. Materials and Methods

**Figure 1:**
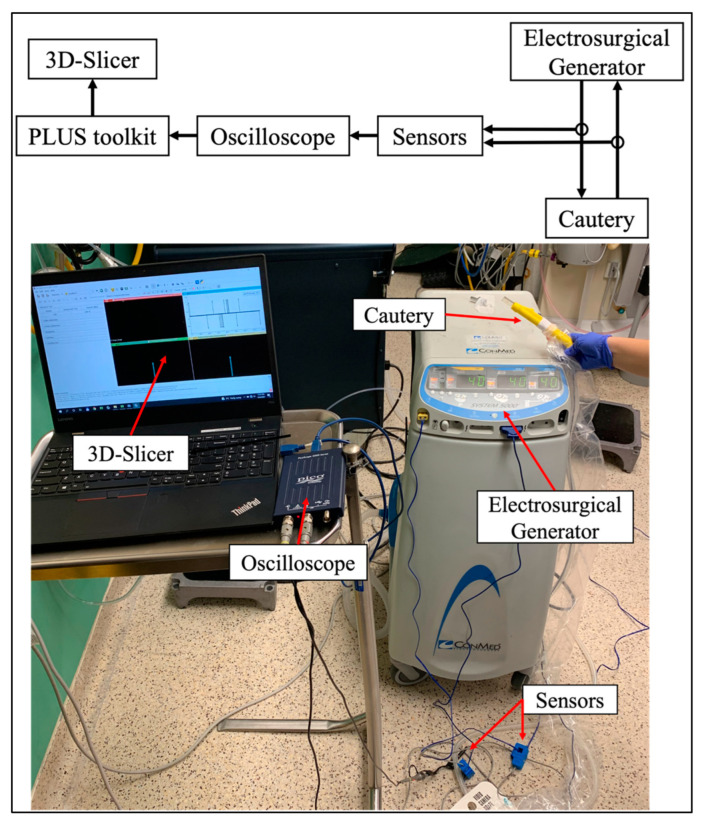
Schematic of the experimental set-up for automated, sensor-driven data collection on electrocautery status (**top**) and experimental set-up in the operating room for ex viva data collection sessions (**bottom**).

To identify the energy event and settings of the cautery, we made incisions on ex vivo tissue samples and detected the changes in the current of the cautery using a non-invasive current sensor and oscilloscope (Figure 1). The voltage signal was streamed into the end-user application via the OpenIGTLink communication protocol by the PLUS toolkit. PLUS is an open-source software library that enables applications to communicate effectively with hardware and underpins a wide variety of medical device research [24]. The signal output was sent to the end-user application, 3D Slicer. 3D Slicer is an open-source software for medical computing, commonly used in surgical navigation and CAS [25]. We implemented an open-source 3D Slicer module that used machine learning to automatically detect and display surgical incisions, the cautery’s mode and cautery settings with no change to the surgical workflow. The oscilloscope and current sensors do not interfere with the surgical workflow and can be attached to a wide variety of surgical power tools. PLUS supports a variety of devices and converts data to a standard OpenIGTLink message format. 

### 2.1. Experimental Set-Up

Two cautery devices were used in this study: a Valley Lab Force FX C (Avante Health Solutions, IL, USA) and a ConMed System 5000 (ConMed Corporation, New York, NY, USA). Each cautery device was tested at three different power levels: at 30 W, 35 W, and 40 W, for both cut and coagulate modes. These energy levels were selected because they form standard practices during surgical operations. We tested three different ex vivo tissue specimens for each cautery device and wattage including chicken, porcine, and bovine. Building on our previous work, we attached an SCT-013 current sensor (YHDC, Madrid, Spain) to the live and return electrodes of the cautery [8,22]. The two sensors were connected to a PicoScope P2204A USB oscilloscope (Pico Technologies, St Neots, UK) that digitized the electrical signals. Voltage data as a relative estimate of current in the cautery leads was streamed from the oscilloscope into 3D Slicer via the PLUS toolkit. Data was streamed into 3D Slicer at a rate of 20 Hz with 3900 samples in each data packet.

### 2.2. Cautery State and Energy Level

**Figure 2:**
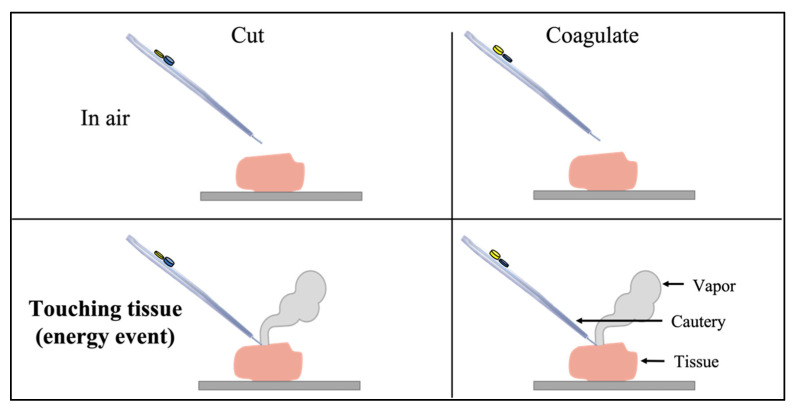
Cautery states in “on” mode, which need to be controlled differently depending on the outcome of the mass spectrometry.

The cautery has five different states: off, cut mode with the blade in air, cut mode with the blade touching tissue (energy event), coagulate mode with the blade in air, and coagulate mode with the blade touching tissue (energy event) (Figure 2). The start of a surgical incision is defined as the cautery state transition from off, cut-in-air, or coagulate-in-air, to being in cut-touching-tissue or coagulate-touching-tissue. The end of an incision is the reverse transition. Therefore, we need to detect all five cautery states.

The three energy levels that are most commonly used in surgery also need to be automatically detected. We detected these energy levels across both cautery machine models. We only need to detect the energy levels when the cautery was in the air state, because a surgeon can turn on the cautery prior to the beginning of an operation. This allowed us to determine the energy level of the cautery prior to the operation beginning. The energy level setting was rarely changed during surgery.

### 2.3. Data Acquisition

**Figure 3:**
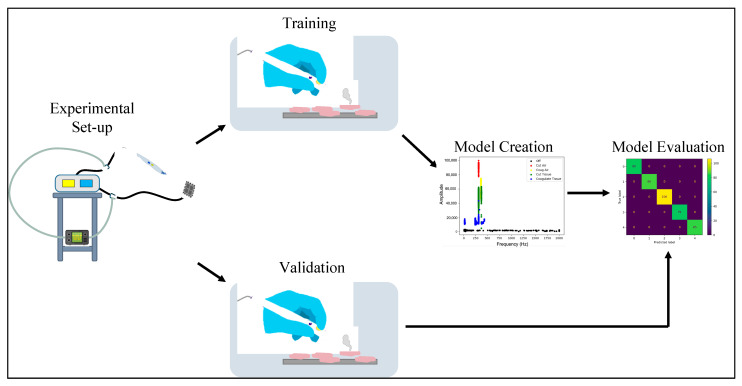
Overall workflow: experimental set-up, data acquisition, model training, validation. Mass spectrometry of tissue smoke is used to determine the classification of the tissue subjected to electrocautery.

We sampled the waveform of the cautery’s current over 18 different test set-ups: two cautery devices, three power settings, and three tissue types (Table 1). Current sampling was conducted for these parameters in cut-tissue and coagulate-tissue states only. For cautery states off, cut-air and coagulate-air, the tissue sample does not change the waveform, thus the current was not sampled in these states. Each tissue test contained 120 incisions for both cut and coagulate modes. Each incision was approximately 1 sec in length. For cautery state detection, validation was done with 8-fold cross-validation (Table 2). Each fold left out all data from each testing variable: tissue (chicken, porcine, or bovine), power (30 W, 35 W, or 40 W), and machine (ConMed or Valley Lab) (Manufacturer, HeadO). Energy level detecting was done using the air states for three power levels (30 W, 35 W, and 40 W) for each cautery device (ConMed or Valley Lab). The training and validation data sets contained distinct tissue samples to ensure no overlap in model training. The overall workflow is visualized in Figure 3.

**Figure 4:**
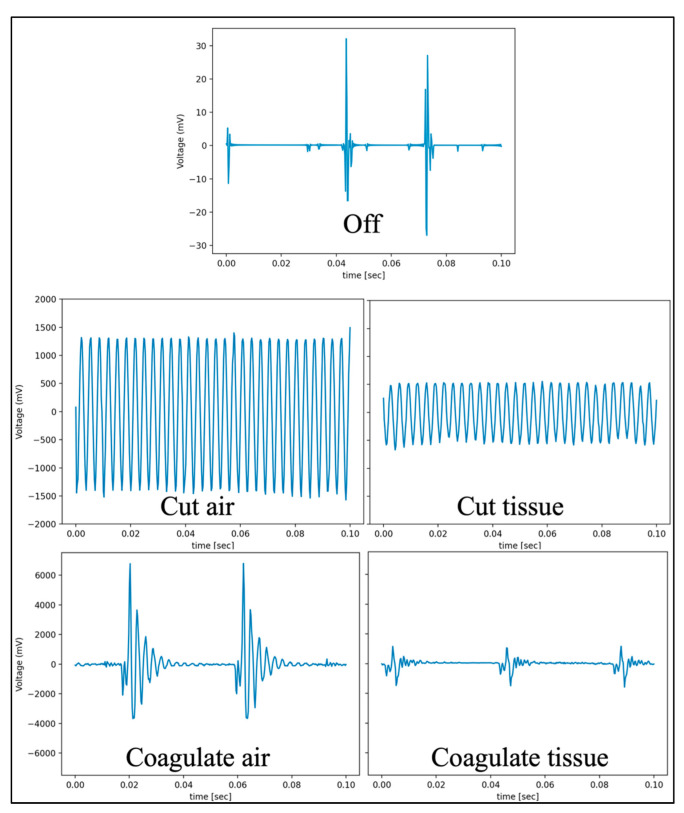
Current samples from the live cautery wire for each cautery state on the Valley Lab ESU. The voltage patterns and values measured for each cautery mode differed significantly.

Data samples were collected in packets, every 50 milliseconds (ms). Data samples were streamed into the 3D Slicer at a rate of 20 Hz. Each data sample contained 3900 data points of the cautery’s current resulting in a sampling rate of 78 kHz. The final dataset contained a total of 69,532 data samples from the cautery’s states. For tissue testing, we recorded voltages from the current sensors (Figure 4) and ran the classifier on each cautery state. 

### 2.4. Model Training

Fast Fourier Transform (FFT) was applied to the voltage signals. FFTs were down sampled to 200 frequency bands and clustered using Principal Component Analysis (PCA) in selecting features for classification and for visualization of data classes. Features from the FFT were used to train a Support Vector Machine (SVM) and a Random Forest Classifier (RFC). As an input into the SVM and RFC, we extracted the maximal intensity and corresponding frequency from the FFT output for each cautery state. We also built machine learning models trained on the first five principal components. To detect the cautery state, we used a leave-one-out 8-fold cross validation for each variable: tissue (chicken, porcine, or bovine), power (30 W, 35 W, or 40 W), and machine (ConMed or Valley Lab). To detect the cautery’s power level (30 W, 35 W or 40 W), we used cautery mode (cut vs. coagulate) in air. Voltage data analysis and machine learning models were implemented as an open-source module for the 3D Slicer application supporting real-time analysis (https://github.com/SlicerIGT/LumpNav.git (accessed on 1 February 2022)). Data for validation of our proposed methodology can be accessed upon request. This allows for other research groups to easily implement similar solutions on their own, or to replicate our results for validation or benchmarking. Objectively comparing the machine-learning algorithms’ outcome has become an increasingly important challenge for CAS systems [26].

## 3. Results

The average SVM and RFC validation classification accuracy for detecting cautery states using maximal intensity and corresponding frequency, and principal components are presented in Table 3 and Table 4, respectively. The average accuracy scores for detecting the energy level are shown in Table 5 and Table 6, respectively. The top performing model was the RFC for both cautery devices. The SVM still had good accuracy. The results for each machine were similar. 

**Figure 5:**
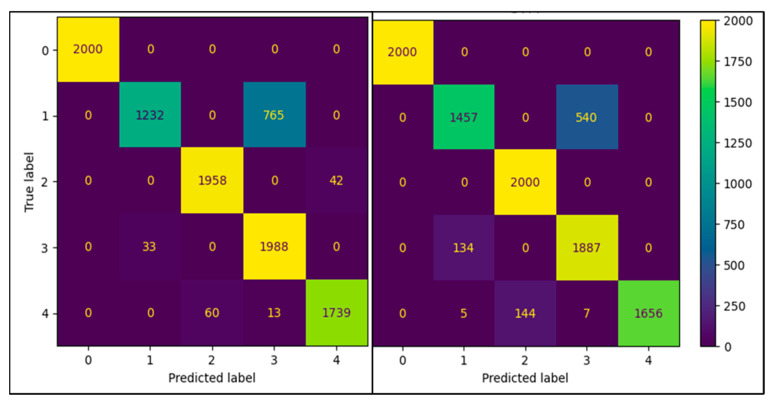
Correlation matrix for leave-out 30 W for RFC (**left**) and SVM (**right**) using the maximal intensity and corresponding frequency.

**Figure 6:**
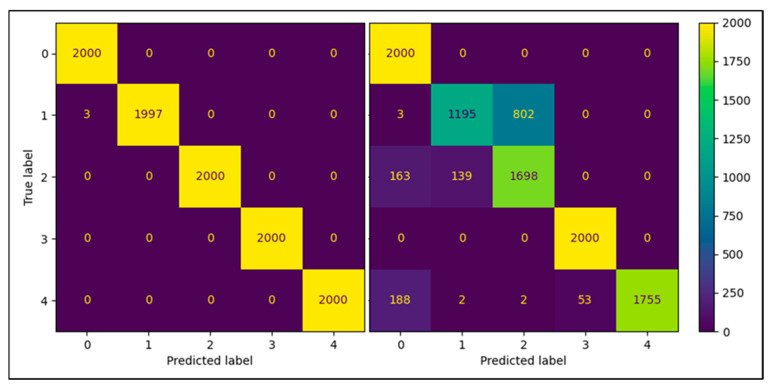
Correlation matrix for leave-out 30 W for RFC (**left**) and SVM (**right**) using the first five principal components.

**Figure 7:**
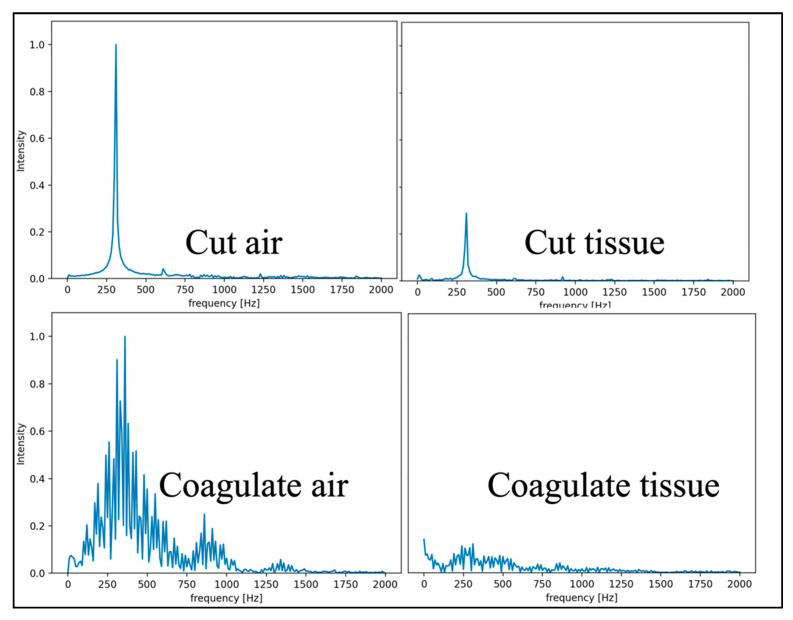
FFTs (relative intensive vs. frequency) when Valley Lab cautery is on.

**Figure 8:**
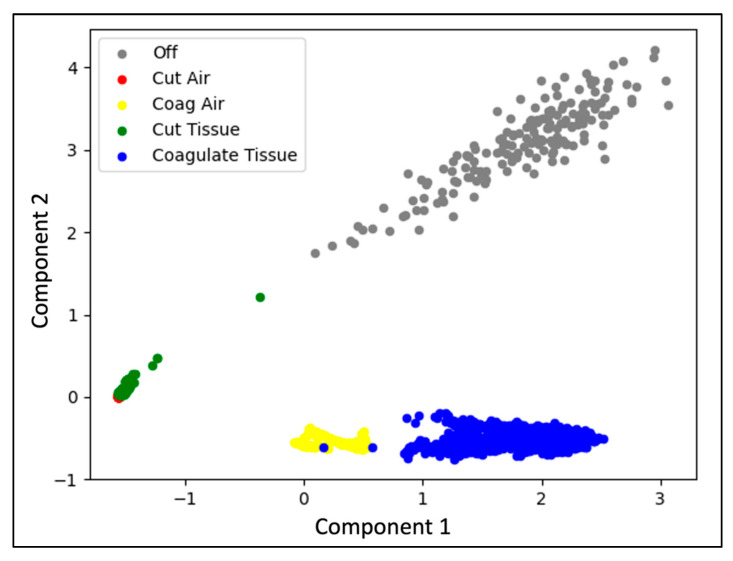
Principal Component Analysis of five cautery states for collection with Valley Lab cautery device, set to 40 W power excising bovine tissue.

**Figure 9:**
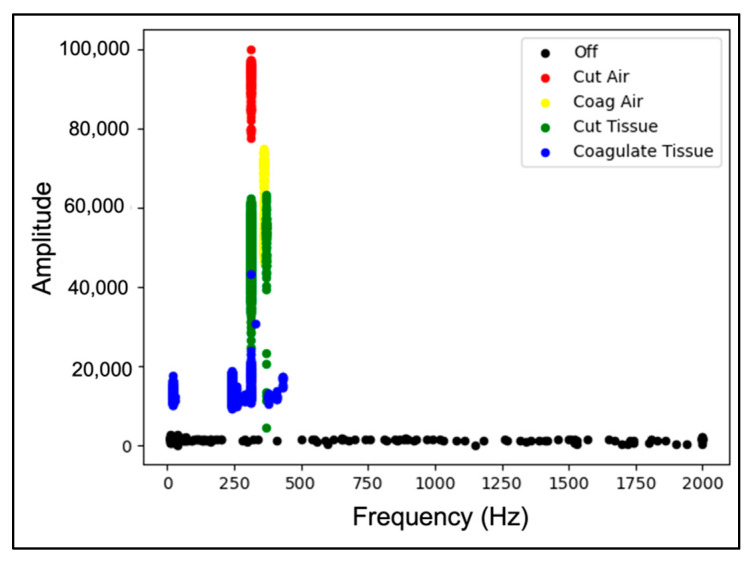
SVM features used to separate cautery signal for collection with Valley Lab cautery device set to 40 W power excising bovine tissue.

The standard models were used for both the SVM and RF classifiers based on [27]. An example correlation matrix for the leave-out 30 W fold can be seen in Figure 5 and Figure 6. Frequency domain transformations for each cautery state are displayed in Figure 7. Principle Component Analysis on the frequency bins of each sample are seen in Figure 8. FFT features used to train the SVM and RFC are seen in Figure 9.

The RFC and SVM performed comparably on the leave-out tissue and power level sets. On average, the top performing classifier was the RFC for the leave-out sets on tissue (98.93% accuracy) and power (97.22% accuracy). For the cautery device leave-out set, the top performing model was the SVM (57.03% accuracy). The models performed the best on the leave-out tissue set. On the leave-out power set, the models performed acceptably. On the cautery device leave-out set, the models did not perform well.

The RFC performed better than the SVM for the majority of the leave-out sets. On average, the RFC was the top performing model for the leave-out sets on tissue (98.52% accuracy), power (97.98% accuracy), and cautery device (86.00% accuracy). The models using the principal components were more effective in classifying the cautery state regardless of ESU type.

The top performing model was the RFC for both cautery devices. The SVM still had good accuracy. The results for each machine were similar. 

The top performing model was the RFC for both cautery devices. The SVM performed poorly when classifying the power level.

Two of the confusion matrices are displayed in Figure 5 and Figure 6 for the leave-out 30 W power set. Both models performed well. As seen above, the model struggled the most with separating the cut air and cut-tissue cautery states (classes 1 and 3, respectively). 

Figure 8 displays a PCA plot for the Valley Lab-40 W-bovine experiment. There is separation between each of the cautery states. The narrowest margin of separation in states is between cut air and cut tissue. This aligns with the results seen in the confusion matrix and is consistent across all models, confusion matrixes, and PCAs for each of the leave-out sets.

Figure 9 visualizes the frequency features used for training the SVM and RFC models. The maximal intensity and corresponding frequency were plotted against each other and annotated based on the cautery state. For each feature, there is clear separation for each cautery state. Some overlap in feature space occurs between cut-air and cut-tissue states. This is representative of the confusion matrixes and PCA plots for each model.

**Figure 10:**
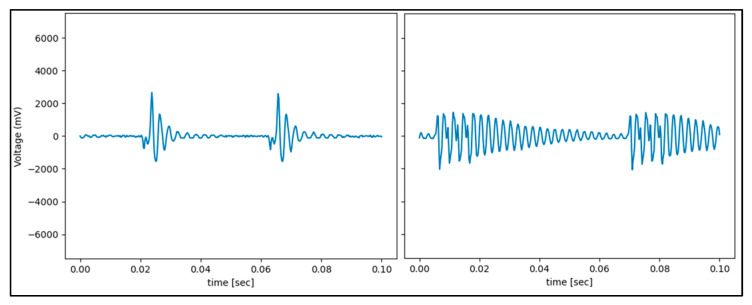
Coagulate Valley Lab (**left**) vs. ConMed (**right**).

Consistent across each model was the lower classification accuracy when identifying cut-air vs. cut-tissue states. This is because the differences in electrical signal patterns are less pronounced. As seen in the PCA plot, the cut-air and cut-tissue data points have less separation. Additionally, each ESU has different frequencies associated with each cautery state (Figure 10). As a result, the model struggled to classify different ESU data sets.

The differences between the Valley Lab and ConMed cautery device signal can be seen in Figure 10. Both the frequency and intensity of the signal were significantly different across the devices.

## 4. Discussion

Our results show that each cautery state can be classified with high accuracy regardless of the different tissue types and energy level parameters altered during operations. The machine learning models were also able to detect the energy level of the cautery. To our knowledge, this is the first study that shows automatic detection of both the energy events and the settings of a cautery, which could be an enabling feature towards automated electrocautery tool management, or an additional safety feature. We developed a sensor-based solution that is low-cost, easy to implement, and most importantly does not require any changes in the surgical workflow. We implemented the methods in an open-source software to allow a wider research community to collect data, train and test their machine learning models, and implement and benchmark their solutions. Our study demonstrates the feasibility of using the cautery state automatically to identify when surgeons make incisions during their operations.

Machine learning provided a rapid and effective way to classify our data from the non-invasive current sensors. Simpler classification methods, such as hardware electrical solutions provided by additional circuits in the electrosurgical devices, may be used in future solutions; however, those solutions would require a long approval process, because they would change the closed commercial electrosurgical units. The machine learning models tested in our experiments performed poorly when one cautery device was left out from training; an expected result based on the different frequencies and waveforms between the cautery machines (Figure 10). To overcome this, separate models were built for each cautery machine. 

For each leave-out set, four machine learning models were built: an RFC and SVM using the maximum intensity and corresponding frequency, and an RFC and SVM using the first five principal components from the PCA. The models using the principal components performed better on the leave-out sets for power level and ESU. The maximum intensity and corresponding frequency features were more effective at classifying tissue types. We found that the RFC consistently outperformed the SVM when using the principal components. One reason for this may be that RFC performs better with higher dimensionality data. In light of the principal components’ accuracy results, we believe the maximal intensity and corresponding frequency are more robust features to use for classification. Detecting the cautery state regardless of different tissues will be the primary use case for a future system. This is because surgeons will be cutting through different tissue types during their operation and maintaining a relatively constant energy level. More cautery state classifications will be made on multiple tissue types than energy levels. To ensure our model’s robustness throughout the operation, we must be able to detect the energy event regardless of tissue types. In future machine learning models, using a combination of both PCA and maximal intensity and corresponding frequency may produce strong accuracy results across all leave-out sets. 

This study provides a key step towards sensor-integrated, fully navigated intra-operative mass spectrometry tissue analysis for breast-conserving surgeries. By combining the timepoints of energy events during surgical incisions with position tracking, we can locate tissue and tumor classifications detected by REIMS. Integrating these methods allows us to classify the cautery state multiple times during each REIMS classification, as REIMS requires at least 1 sec of sampling time for one tissue classification, whereas our cautery state classifier samples at 20 Hz. Using majority voting on the cautery state classifier may improve the accuracy of our model in clinical practice. We can combine 20 cautery state classifications to improve our detection method. Detecting the cautery state also allows us to analyze differences in mass spectra signatures because the cautery’s mode changes the mass spectra of REIMS [19]. By identifying variation in the mass spectra, we can improve the accuracy of REIMS models. Our experimental device set-up can be implemented on a wide variety of electrosurgical devices. Some devices that may require detecting the energy event include RF ablators and vessel sealers. In ablation, tissue contact is essential to destroy dysfunctional tissue [28,29]. Maintaining effective and stable tissue contact during ablation would minimize complication risk resulting from delivery of energy to nearby tissue structures [19]. There is a need for a safety system that monitors an ablator’s energy which would inform clinicians when their ablator loses contact with surrounding tissue to reduce damage to surrounding tissue [30,31,32,33]. The feasibility to detect energy levels using our methodology is validated with our experiments.

Identifying the cautery state can also improve surgical workflow analysis, mass spectra analysis, and tissue characterization. It is important to identify the proper location of incisions—a key step in an operation—in relation to anatomy and blood supply [34]. Automatically locating surgical incisions may be especially important when incisions must be made in obstructed views, when there is shifting in tissues during the operation, and to ensure minimal destruction to surrounding anatomical structures. Since inflammation and immune responses are correlated with the length of surgical incisions [35], patient infection rates may decrease by providing surgeons with more incision length and location information.

We are limited in this study by a small sample size, which may limit our ability to detect changes in the current signal due to variation in patient tissue. We did not use any patient tissue to train our models. Each patient has slightly different levels of cellular resistance. Using real-patient tissue may well impact the current signal. To mitigate this, we tested three different types of animal tissues. During surgical operations, the location of the grounding pad varies, which may affect the cautery’s current flow. The location of the grounding pad was not varied during data collection. The grounding pad location may impact the resulting cautery current signal. Additionally, we did not use any data from a surgical operation. In the future, it would be important to collect data from a breast cancer surgery and classify the current signal. Finally, the location of the current sensors was not varied during the experimental set-up. Altering the distance from the current sensor to the ESU may impact the intensity of the signal due to electromagnetic interference. It would be important to evaluate the interference of other electrosurgical tools used in the operating room; one solution would be to insulate the current sensors to eliminate the interference. The parameters listed above may impact the model’s ability to effectively classify the cautery state. 

Additionally, our model may require re-training, depending on the ESU selected. Our model identifies the cautery state using the ESU frequency, a factor that varies between cut and coagulate modes across ESUs. Therefore, one model may be insufficient to classify the cautery state using all ESUs, and a list of models for each ESU may need to be created. The models were able to separate cut-air from cut-tissue cautery states; however, these were the classes that the model struggled the most to separate. One of the reasons may be because there are fewer changes in frequency seen between these states. In the future, it may be effective to model the noise separately in the cautery’s current signal to better identify the cautery states. We did not evaluate the spatial accuracy of our model using the location of surgical incisions. A next step includes evaluation of the model with an EM tracking system. By pairing the navigation system with the model, we can determine the accuracy of locating surgical incisions, which would be an important clinical support function.

## 5. Conclusions

To our knowledge, this is the first study that investigates the option to automatically detect the energy events and settings of an electrocautery device, including the cautery’s mode (cut vs. coagulate), and the energy level. The procedure described in this article is a robust and automatic method to make these detections, experimented on two cautery devices, which classifies cautery states with an average accuracy of 95.56% across different tissue types and energy levels. This was completed using an open-source software to allow other researchers to collect data, and train and test their models to be implemented. Our results demonstrated the feasibility of implementing a low-cost solution, which is easy to implement and does not disrupt the surgical workflow. In the future it will be important to increase the sample size of in vivo tissue testing in addition to investigating the effects of viscoelasticity on locating tissue classifications. Our methods can be applied to a variety of surgical procedures. In robotic and telesurgery, updating surgeons when their electrosurgical tool touches patient tissue may provide increased feedback beyond visual feedback from a camera or scope. In RF ablation, detecting the energy event and level of the ablator may help surgeons ensure they completely destroy dysfunctional tissue. By combining the time of a surgeon’s incisions with position tracking, we can locate tumor signals detected by the REIMS. This study provides a key step towards fully navigated intra-operative mass spectrometry tissue analysis, which may see its clinical application for breast-conserving surgeries among others.

## Figures and Tables

**Table 1:** Testing protocol for the cautery states for each of the 18 tests set-ups.

	Chicken		Porcine		Bovine	
30 W	ConMed	Valley Lab	ConMed	Valley Lab	ConMed	Valley Lab
35 W	ConMed	Valley Lab	ConMed	Valley Lab	ConMed	Valley Lab
40 W	ConMed	Valley Lab	ConMed	Valley Lab	ConMed	Valley Lab

**Table 2:** Overview of 8-fold cross-validation (grey = validation, white = training).

Chicken	Liver	Pork	30 W	35 W	40 W	ConMed	Valley Lab
Chicken	Liver	Pork	30 W	35 W	40 W	ConMed	Valley Lab
Chicken	Liver	Pork	30 W	35 W	40 W	ConMed	Valley Lab
Chicken	Liver	Pork	30 W	35 W	40 W	ConMed	Valley Lab
Chicken	Liver	Pork	30 W	35 W	40 W	ConMed	Valley Lab
Chicken	Liver	Pork	30 W	35 W	40 W	ConMed	Valley Lab
Chicken	Liver	Pork	30 W	35 W	40 W	ConMed	Valley Lab
Chicken	Liver	Pork	30 W	35 W	40 W	ConMed	Valley Lab

**Table 3:** Average accuracy from cross-validation detecting cautery state using maximal intensity and corresponding frequency.

Leave-Out	Accuracy			Average Accuracy
Tissue	Chicken: SVM = 98.34% RFC = 98.64%	Porcine: SVM = 98.74% RFC = 98.91%	Bovine: SVM = 99.33% RFC = 99.24%	SVM = 98.82% RFC = 98.93%
Power	30 W: SVM = 95.31% RFC = 97.03%	35 W: SVM = 92.98% RFC = 97.75%	40 W: SVM = 96.88% RFC = 96.89%	SVM = 95.06% RFC = 97.22%
Cautery Device	ConMed: SVM = 68.77% RFC = 29.83%	Valley Lab: SVM = 45.29% RFC = 27.19%		SVM = 57.03% RFC = 28.51%

**Table 4:** Average accuracy from cross-validation detecting cautery state using the first five principal components.

Leave-Out	Accuracy			Average Accuracy
Tissue	Chicken: SVM = 86.21% RFC = 95.56%	Porcine: SVM = 92.86% RFC = 100%	Bovine: SVM = 93.24% RFC = 100%	SVM = 90.77% RFC = 98.52%
Power	30 W: SVM = 87.70% RFC = 97.46%	35 W: SVM = 86.14% RFC = 99.86%	40 W: SVM = 87.80% RFC = 96.61%	SVM = 87.21% RFC = 97.98%
Cautery Device	ConMed: SVM = 38.46% RFC = 89.18%	Valley Lab: SVM = 25.25% RFC = 82.81%		SVM = 31.86% RFC = 86.00%

**Table 5:** Average accuracy detecting the energy level for each cautery using maximal intensity and corresponding frequency.

	Accuracy	
Cautery Device	ConMed: SVM = 70.08% RFC = 99.05%	Valley Lab: SVM = 75.74% RFC = 99.93%

**Table 6:** Average accuracy detecting the energy level for each cautery using the first five principal components.

	Accuracy	
Cautery Device	ConMed: SVM = 32.01% RFC = 83.41%	Valley Lab: SVM = 25.05% RFC = 100%

## Data Availability

Software from this study is contained in the GitHub repository: https://github.com/SlicerIGT/LumpNav.git (accessed on 1 July 2022) and data is available upon request.
